# PROGNOSTIC FACTORS FOR LEFT COLECTOMY FOR COLON CANCER: A TEN YEARS EXPERIENCE OF A SINGLE UNIVERSITY INSTITUTION

**DOI:** 10.1590/0102-6720201700020006

**Published:** 2017

**Authors:** Sergio Carlos NAHAS, Caio Sergio NAHAS, Leonardo Alfonso BUSTAMANTE-LOPEZ, Rodrigo Ambar PINTO, Carlos Frederico Sparapan MARQUES, Fabio Guilherme CAMPOS, Ivan CECCONELLO

**Affiliations:** 1 Gastroenterology Department, Clinic Hospital, University of São Paulo, São Paulo, SP, Brazil.

**Keywords:** Colonic neoplasms, Lymph nodes, Surgical procedures, Colorectal surgery

## Abstract

**Background:**

Colorectal cancer is the third most common cancer in the world. In Brazil, it is the leading cause of cancer in the gastrointestinal tract.

**Aim:**

To evaluate the preoperative, perioperative, and postoperative risk factors for recurrence and overall survival of patients with left colon cancer operated during a ten-year period.

**Methods:**

Patients with left colon cancer surgically treated underwent clinical preoperative workout and cancer staging. The following factors were studied: gender, age, tumor location, T stage, lymph node yield, N stage, M stage, histological type, and tumor differentiation. It was analyzed the influence in five-year overall survival.

**Results:**

A total of 173 patients underwent left colectomy for colon cancer. There was a slight predominance of male gender with 50.9%. The mean age was 60.8 years old. Fifteen (8.7%) tumors were located at splenic flexure, 126 (72.8%) at sigmoid colon, and 32 (18.5%) at descending colon. The median length of hospital stay was seven days. Mean survival was 47.5 months. At 60 months seven patients (4%) lost follow-up, 38 patients (21.9%) deceased and 135 patients (78%) were alive. Overall survival time was 48 months.

**Conclusion:**

Advanced stages (T3-T4, N+ and M+) were the only factors associated with poor long term survival in left colon cancer.

## RESUMO

**Racional:**

O câncer colorretal é o terceiro câncer mais comum no mundo. No Brasil é a principal causa no trato gastrointestinal.

**Objetivo:**

Avaliar os fatores de riscos pré, peri e pós-operatório para recorrência e sobrevida global de pacientes com câncer de cólon esquerdo operado durante um período de dez anos.

**Métodos:**

Os pacientes com câncer de cólon esquerdo operados foram retrospectivamente avaliados. Os seguintes fatores foram estudados: gênero, idade, localização do tumor, estádio T, número de linfonodos, estágio N, estágio M, tipo histológico e diferenciação tumoral. Foi analisada a influência desses fatores na sobrevida global de cinco anos.

**Resultados:**

173 pacientes foram submetidos à colectomia para câncer de cólon esquerdo. O gênero masculino predominou com 50,9%. A média de idade foi de 60,8 anos. Quinze (8,7%) tumores foram localizados no ângulo esplênico, 126 (72,8%) no sigmóide e 32 (18,5%) e no descendente. A média do tempo de internação foi de sete dias. A sobrevida média foi de 47,5 meses. Aos 60 meses sete doentes (4%) perderam o seguimento, 38 (21,9%) faleceram e 135 (78%) estavam vivos. O tempo de sobrevida global foi de 48 meses.

**Conclusão:**

Os estádios avançados (T3-T4, N e M+) foram os únicos fatores associados à menor sobrevida em longo prazo em câncer de cólon esquerdo.

## INTRODUCTION

Colorectal cancer (CRC) is the third most common cancer in the world^20^. In Brazil it is the leading cause of cancer in the gastrointestinal tract, and the third most prevalent cancer. It is estimated that 2:100 000 people die each year in Brazil affected by CRC^12^. Radical surgery involves the removal of the intestinal segment involved by the tumor, which must include an adequate margin of resection and complete loco regional lymphadenectomy^23^. Complete removal of the tumor along with the major vascular pedicle and the lymphatic drainage, either by laparotomy or laparoscopy is the best curative option for patients with localized colonic cancer^8^ ^,^ ^14^ ^,^ ^15^. Left colectomy for the cancer localized at the descending and sigmoid colon is the procedure of choice for complete oncologic resection and favorable prognosis^3^.

Survival differences are generally explained by the understaging of the disease because of suboptimal cancer surgery or inadequate pathological examination of the specimen. Accurate staging of colon cancer is principal to satisfactory oncological outcome. The necessity of a sufficient lymph node yield, adequate margins, and standardized operative techniques has been established^18^.

Despite the surgical treatment of left colon cancer has been well standardized, even in the better services it is not exempt of postoperative complications that may occur between 3.6 to 23%^7^. Furthermore, despite the complete oncologically surgery, the survival rates are 65-83%^3^ ^,^ ^7^.

The aim of the present study was to evaluate the pre-, peri- and postoperative risk factors for recurrence and overall survival of patients with left colon cancer operated during a ten-year period in a universitary service.

## METHODS

Patients with left colon cancer referred and surgically treated at the Service of Colon and Rectal Surgery of the *Hospital das Clínicas, School of Medicine, Universiy of São Paulo, Brazil,* from 2002-2012 were retrospectively evaluated based on a prospective database. In this period, 1219 patients with colorectal adenocarcinoma were treated. Of these, 566 patients had colon cancer, of which 173 underwent left colectomy. Patients with incomplete data, synchronous cancers, or with benign disease were previously excluded from the analysis.

All patients underwent clinical preoperative workout and cancer staging including laboratory exams and imaging (computed tomography of the thorax, abdomen and pelvis). Left colectomy was performed by laparoscopic or conventional laparotomy approaches. The surgical procedures respected the oncologic principles, which included en-bloc resection, adequate lymphadenectomy, high ligation of vascular pedicles and tumor-free resection margins.

The following factors were studied: gender, age, tumor location, wall invasion depth (T stage), lymph node yield, lymph node status (N stage), presence of distant metastases (M stage), histological type, and tumor differentiation. It was analyzed the influence of these factors in five-year overall survival.

Bowel preparation was performed on the day before surgery. One liter of 10% mannitol solution was used for cases scheduled as open procedure, while 90 ml of 10% sodium phosphate solution was used for laparoscopic cases to prevent bowel gaseous distension. Intravenous antibiotic prophylaxis was performed 1 h before the operation to anesthetic induction with second or third generation cephalosporin alone or combined with metronidazole, and maintained postoperatively for up to 36 h. All patients received mechanical and chemical thromboembolic prophylaxis.

The procedure standardized for tumors located at splenic flexure, descending colon, or sigmoid and rectosigmoid colon in our institution was the left colectomy, including a medial-to-lateral approach by laparoscopy or lateral-to-medial procedure by laparotomy. Splenic flexure was routinely mobilized, and inferior mesenteric artery and vein were ligated in their origins. A tension free end to end colorectal anastomosis was performed after excising the specimen with adequate margins (minimum 10 cm proximal and 5 cm distal margins).

### Statistical analysis

The SPSS software 20.0® was used for statistical analysis. Patient and tumor characteristics were described with estimate measures (mean, standard deviation, and median, minimum and maximum) for quantitative variables. Absolute and relative frequencies were described for qualitative variables (Kirkwood and Sterne, 2006). Overall survival was estimated according to the characteristics of interest using the Kaplan-Meier function. For the significant variables for overall survival at the univariate analysis the Cox multiple regression model was applied. The tests were performed at a significance level of 5%.

## RESULTS

A total of 173 patients underwent left colectomy for colon cancer. There was a slight predominance of male gender with 50.9%. The mean age was 60.8 (22-87) years old. Fifteen (8.7%) tumors were located at splenic flexure, 126 (72.8%) at sigmoid colon, and 32 (18.5%) at descending colon. The median length of hospital stay was seven days (5-60). At 60 months seven patients (4%) lost follow-up, 38 patients (21.9%) deceased and 135 patients (78%) were alive. Overall survival time was 48 months (Table 1).

**TABLE 1:** Patients characteristics

Variable	Description (n=173)
Age (years)	
Man (SD)	60.8(13.2)
Median (min; max)	63 (22; 87)
In-hospital time (days)	
Mean (SD)	
	14 (5; 60)
Survival time (months)	
Mean (SD)	47.4 (34.6)
Median (min, max)	40 (0; 124)
Gender, n (%)	
Female	85 (49.1)
Male	88 (50.9)
Site, n (%)	
Splenic flexure	15(8.7)
Sigmoid colon	126 (72.8)
Descending colon	32(18.5)
T stage, n (%)	
T1	15(8.7)
T2	19(11.0)
T3	111 (64.2)
T4	28(16.2)
N stage, n (%)j	
NO	101 (58.4)
N+	72 (41.6)
M, n (%)	
Negative	167 (96.5)
Positive	6 (3.5)
Stage, n (%)	
1	25(14.5)
II	78 (45.1)
III	64 (37.0)
IV	6 (3.5)
Lymph node, n (%)	
Less than 12	32(18.5)
12 or more	141 (81.5)
Histological type, n (%)	
Nonmucinous	163 (94.2)
Mucinous	10(5.8)
Tumor differentiation, n (%)	
Well	163 (94.2)
Poorly	10(5.8)

Out of 173 patients, one hundred eleven (64.2%) were stage T3. Among those patients, 64 (57.6%) were T3N0, 26 (23.4%) were T3N1, and 21 (18.9%) were T3N2. 139 patients (80.4%) had T3 or T4 tumors, and 72 (41.6%) had lymph node involvement.

The average number of lymph nodes retrieved per patients was 23. Out of 28 patients classified as T4, 11 (39.2%) had no lymph node involvement, six (21.4%) were N1, and 10 (3.5%) were N2; 163 (94.2%) had non-mucinous tumors; 163 (94.2%) had moderated or well differentiated tumors. 

**FIGURE 1:**
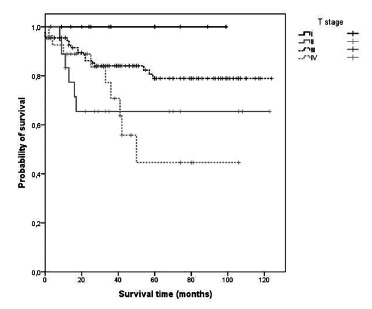
Kaplan-Meier survival estimates for patients with left colon cancer according with T stage

**FIGURE 2:**
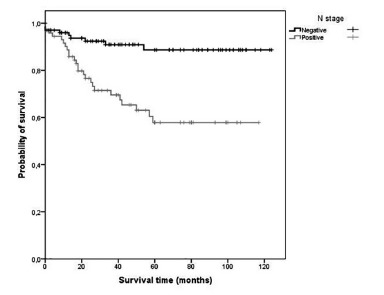
Kaplan-Meier survival estimates for patients with left colon cancer according with N stage

Table 2 shows that advanced T and final stage are associated with decreased overall survival (p = 0.023 and p <0.001 respectively). (Figure 1) Also, positivity of N status (Figure 2) and M status (Figure 3) led to reduction in patient survival time (p <0.001 and p <0.001 respectively). Our 30 days mortality was 2.9% (5 patients), and 5 years mortality was 19.7% (34 patients). Cancer recurrence was responsible for mortality in 20 (52.6%) of these patients. Besides that, out of 34 patients who died within 5 years, 32 (94.1%) were T3, T4 or N positive. 

**TABLE 2:** Survival analysis of patients according to characteristics of interest

Variable	Mean estimate	95% Cl		HR	95% Cl	n of Events	Total n	%	p
		Lower	Upper		Lower	Upper				
Gender										0.500
Female	90.49	79.98	101.00	1.00			18	85	21.2	
Male	101.83	92.07	111.60	0.79	0.41	1.56	16	88	18.2	
Age (years)										0.919
40 or less	86.80	63.70	109.90	1.00			3	15	20.0	
41 to 60	100.28	87.93	112.63	0.87	0.24	3.11	11	62	17.7	
More than 60	93.75	84.40	103.11	1.01	0.30	3.40	20	96	20.8	
Site										0.311
Flexure splenic	86.83	66.65	107.01	1.00			3	15	20.0	
Sigmoid colon	100.70	92.36	109.03	0.93	0.28	3.10	22	126	17.5	
Descending colon	88.07	68.59	107.56	1.67	0.45	6.19	9	32	28.1	
T stage										0.023
I	99.00	99.00	99.00	1.00			0	15	0.0	
II	84.83	60.02	109.64	#			6	19	31.6	
III	102.96	94.44	111.49	#			19	111	17.1	
IV	65.44	46.13	84.74	#			9	28	32.1	
N stage										<0.001
Negative	112.32	105.05	119.58	1.00			9	101	8.9	
Positive	78.38	66.48	90.28	4.04	1.88	8.65	25	72	34.7	
M										<0.001
Negative	101.13	93.82	108.44	1.00			30	167	18.0	
Positive	24.46	14.53	34.39	5.74	1.97	16.76	4	6	66.7	
Stage										<0.001
I	112.46	98.52	126.41	1.00			2	25	8.0	
II	109.07	99.97	118.18	1.30	0.28	6.01	9	78	11.5	
III	83.67	71.36	95.97	3.52	0.82	15.10	19	64	29.7	
IV	24.46	14.53	34.39	12.14	2.19	67.47	4	6	66.7	
Lymph node										0.203
Less than 12	90.10	71.39	108.80	1.00			9	32	28.1	
12 or more	100.07	92.08	108.07	0.61	0.29	1.31	25	141	17.7	
Histological type										0.167
Non-mucinous	97.76	90.04	105.47	1.00			34	163	20.9	
Mucinous	89.00	89.00	89.00	0.05	0.00	35.98	0	10	0.0	
Differentiation										0.547
Well	99.53	92.02	107.03	1.00			32	163	19.6	
Poorly	65.00	44.50	85.50	1.55	0.37	6.47	2	10	20.0	
Total	98.96	91.53	106.38				34	173	19.7	

Table 3 shows that patients with N+ stage showed risk of death 3.8 times higher than the risk of patients with N0 stage. (Figure 2) In addition, patients with metastatic disease presented a risk of death 3.3 times higher than M negative patients. 

(Figure 1)

**TABLE 3:** Cox regression model for T, N, and M stage

Variable	HR	95% Cl		P
		Lower	Upper	
Age (years)	1.01	0.98	1.03	0.666
Gender (Male)	0.86	0.43	1.71	0.659
T stage (T3 orT4)	0.70	0.29	1.73	0.442
N stage (Positive)	3.86	1.75	8.51	0.001
M (Positive)	3.29	1.10	9.81	0.033
Histological type (Mucinous)	0.00	0.00	.	0.976

The Table 4, shows that patients with stage III or IV had risk of death 3.3 times higher than patients with stage I or II, independently of other factors analyzed

**TABLE 4:** Cox regression model for final staging

Variable	HR	95% Cl		P
		Lower	Upper	
Age (years)	1.01	0.98	1.04	0.557
Gender (Male)	0.84	0.43	1.67	0.623
Stage (III or IV)	3.33	1.61	6.88	0.001
Histological type (Mucinous)	dUO	dUO		0.979

## DISCUSSION

This study was done in a tertiary Brazilian hospital, reference in cancer care. For the purpose of healthcare education, operations were performed by residents in training, always assisted by a member of colorectal surgeon’s staff. The study reflects the importance of colon cancer incidence in the period of ten years in a teaching hospital and analyzes the factors related to survival and mortality of affected patients. It aimed to highlight not only the characteristics and consistency of the procedures performed in an academic institution, but specimen pathological characteristics, immediate postoperative results and long-term follow-up of an average of 60 months.

Differences in clinical presentation, patient demographics, and tumor biology between right and left-sided colon cancers have been reported in the literature^18^. At the present study, authors were interested in identifying factors that might serve as targets for improvement in patient care, which could result in better long-term survival for the patients. From 1219 patients presenting colorectal cancer, 566 patients were operated for colon cancer, and 173 had left side colon adenocarcinoma (splenic flexure, descending and sigmoid colon) and underwent left colectomy which represented 30.6% of colon cancer in a ten year period, which is in agreement with most literature series.^8^ ^,^ ^20^ ^,^ ^23^.

In Brazil the incidence of CRC is variable across regions because of the differences in socioeconomic, political, and cultural differences among them. The incidence has increased in recent decades, mainly in the most developed and industrialized regions. The International Agency for Research on Cancer presents data on the number of deaths caused by colorectal cancer in Brazil; however, these are limited data, because information is not detailed by age, gender, and colon site or location. The Brazilian counsel in 2014^2^, found a predominance of CRC in women with 53.9 % of the new cases. The incidence was 6.4% in female been the second most frequent cancer and 5% in men been the third most common cancer overall. In the present study authors found a slight male predominance dealing only with left colon cancer (51%). The incidence of colorectal cancer in the United States of America is about the same among men and women^20^. Souza et al^22^ analyzed incidence of CRC by gender in Brazil and reported a higher rate for women, despite increased mortality rates for both sexes.

A great majority of our patients were T3 and T4, and the survival in this group was worse than for other stages. This finding agreed with other large series that studied advanced colon cancer^5^ ^,^ ^6^ ^,^ ^16^. A review of Surveillance, Epidemiology, and End Results (SEER) population-based data on colon cancer by the American Joint Committee on Cancer (AJCC) found that T3 have better prognosis than T4, and the number of positive nodes affects prognosis^9^ ^,^ ^23^. At the present study lymph node involvement was also associated with worse prognosis and higher risk of death.

Accurate staging of colon cancer is vital to adequate oncological outcome. The need of a sufficient lymph node yield, adequate margins, and standardized operative techniques have been established, and was described in the present study^20^. The median number of regional lymph nodes harvested was 23 in this study, according to the literature, which may vary between 14 and 32 lymph nodes^4^ ^,^ ^22^. As the extent of lymph node dissection can be considered as an indicator of surgical and pathological quality these findings are supported by other studies demonstrating that the use of a standardized anatomy based upon dissection, and surgical technique was an independent factor influencing cancer specific survival and overall survival in affected patients^10^. The American College of Pathologists and the AJCC, recommend that at least 12 nodes retrieved in colon cancer resection^8^. The majority of patients in the current study who underwent surgery had an adequate nodal assessment. The average of positive lymph nodes in the literature varies from 27 to 36% of the patients in the different series^4^ ^,^ ^8^ ^,^ ^21^. The results of the present study show lymph node involvement in a higher percentage of patients (41.6%), which was directly associated with worse survival rates.

Identification of lymph node metastasis is one of the criterion on which adjuvant treatment is offered. Several staging systems have adopted the number and the location of involved lymph nodes as a fundamental parameter for staging the disease^1^ ^,^ ^17^.

Mucinous adenocarcinoma constitutes 4-19% of CRC worldwide^10^. In this study according with the literature, 6% of patients had mucinous colon adenocarcinomas, with zero mortality. Some studies^3,6,21^ have identified a significant association between mucinous histology and poor prognosis. In this study, that relationship was not recognized, maybe due to the small amount of patients with mucinous histology. Furthermore, patients with non-mucinous cancers were older, had larger tumors and higher T and final stage classification. Hugen et al^11^ noted that the histologic subtype and location of the primary tumor has a strong influence on metastatic pattern in CRC. Their findings suggest that peritoneal and lymph node metastases occurred more often in patients with signet-ring cell carcinoma.

Poor differentiation of tumor has been related to locoregional recurrence of colon cancer^3^ ^,^ ^11^. Although, in this study poor differentiation had not been correlated with worse overall survival or recurrence, it was perhaps due to the small number (6%) of patients with this pathological condition^13^.

Schwenk et al^19^ in a meta-analysis, shows a high variability of, postoperative hospital stay, ranging from 6-12.7 days for conventional operations. In our study the postoperative hospital stay was seven days according to the former study. Maybe for the fact that our patients needs to do all the preoperative exams inside the hospital and for social and economic problems in the country, most of the patients must stay in the hospital for immediate preoperative care. In Brazil, there is a concentration of specialty hospital in large urban places, as a result of the development of the area. Populations from rural areas must travel, for treatment and also to access medical appointments and diagnosis services. Such problems produce delays in diagnosis and treatment of patients.

A recent German study that examined a large number of patients with colon cancer and found a rate of in-hospital mortality for left sided colon cancers of 1.4%, but with a higher proportion of T2 patients. Schwenk et al.^19^ 30 days mortality was 1%, as the others papers have an interval between 2-5%^2^. At the present study 30 days mortality was 3% according to other literature studies, maybe due to a high percentage of advanced stage patients (82% of T3, T4 and N+).

In this paper the overall survival was 78% which can be considered a good result, bearing in mind that the majority of the patients included presented advanced colon cancer stage. The approximate 5-year survival rate for colorectal cancer patients in the United States (all stages included) is 65%^8^. Survival is inversely related to stage: approximate 5-year survival rates are 95% for patients with stage I disease, 60% for those with stage III disease^9^ ^,^ ^20^. The 5-year survival rate of patients is approximately 60% with lymph node metastasis and over 80% without lymph node involvement^8^ ^,^ ^9^. Our overall survival was higher compared to other papers, probably due to the hospital characteristics, which are specialized center with a fully trained staff dedicated on colorectal surgery.

As expected, the results confirmed higher mortality in advanced stage left colon cancer patients. There are important additional factors apart from genetic and molecular changes involved on survival rates, including, histological and differentiation type, tumor stage and lymph node yields. To get better results, there is a need to reinforce colorectal cancer screening in our population in order to prevent these unfavorable conditions at initial diagnosis^12^.

Several limitations have to be acknowledged including those inherent to retrospective analyses. Furthermore, multiple surgeons performed the surgery and multiple pathologists evaluated the specimens.

## CONCLUSION

Advanced stages (T3-T4, N+ and M+) were the only factors associated with poor long term survival in left colon cancer. 
